# Prediction of STN-DBS Electrode Implantation Track in Parkinson's Disease by Using Local Field Potentials

**DOI:** 10.3389/fnins.2016.00198

**Published:** 2016-05-09

**Authors:** Ilknur Telkes, Joohi Jimenez-Shahed, Ashwin Viswanathan, Aviva Abosch, Nuri F. Ince

**Affiliations:** ^1^Clinical Neural Engineering Lab., Biomedical Engineering Department, University of HoustonHouston, TX, USA; ^2^Department of Neurology, Baylor College of MedicineHouston, TX, USA; ^3^Department of Neurosurgery, Baylor College of MedicineHouston, TX, USA; ^4^Department of Neurosurgery, University of ColoradoAurora, CO, USA

**Keywords:** local field potentials, subthalamic nucleus, microelectrode recordings, least mean square algorithm, LDA classification

## Abstract

Optimal electrophysiological placement of the DBS electrode may lead to better long term clinical outcomes. Inter-subject anatomical variability and limitations in stereotaxic neuroimaging increase the complexity of physiological mapping performed in the operating room. Microelectrode single unit neuronal recording remains the most common intraoperative mapping technique, but requires significant expertise and is fraught by potential technical difficulties including robust measurement of the signal. In contrast, local field potentials (LFPs), owing to their oscillatory and robust nature and being more correlated with the disease symptoms, can overcome these technical issues. Therefore, we hypothesized that multiple spectral features extracted from microelectrode-recorded LFPs could be used to automate the identification of the optimal track and the STN localization. In this regard, we recorded LFPs from microelectrodes in three tracks from 22 patients during DBS electrode implantation surgery at different depths and aimed to predict the track selected by the neurosurgeon based on the interpretation of single unit recordings. A least mean square (LMS) algorithm was used to de-correlate LFPs in each track, in order to remove common activity between channels and increase their spatial specificity. Subband power in the beta band (11–32 Hz) and high frequency range (200–450 Hz) were extracted from the de-correlated LFP data and used as features. A linear discriminant analysis (LDA) method was applied both for the localization of the dorsal border of STN and the prediction of the optimal track. By fusing the information from these low and high frequency bands, the dorsal border of STN was localized with a root mean square (RMS) error of 1.22 mm. The prediction accuracy for the optimal track was 80%. Individual beta band (11–32 Hz) and the range of high frequency oscillations (200–450 Hz) provided prediction accuracies of 72 and 68% respectively. The best prediction result obtained with monopolar LFP data was 68%. These results establish the initial evidence that LFPs can be strategically fused with computational intelligence in the operating room for STN localization and the selection of the track for chronic DBS electrode implantation.

## Introduction

Deep brain stimulation (DBS) of the subthalamic nucleus (STN) is an effective therapy for the treatment of the motor symptoms of Parkinson's disease (PD) (Herzog et al., 2004; Hariz, 2012). DBS surgery involves localization of the motor territory of the STN, for permanent implantation of a DBS electrode at this site. Although the exact mechanism of DBS remains to be elucidated, STN stimulation is well-tolerated and improves all of the cardinal symptoms of PD (Levy et al., 2002). However, STN stimulation can result in side effects arising from the spread of stimulation to structures surrounding the STN (Richardson et al., 2009). Moreover, sub-optimal positioning of DBS electrodes accounts for up to 40% of cases of inadequate efficacy of stimulation postoperatively (Okun et al., 2005). Thus, developing quantitative electrophysiological methods to define the optimal site of stimulation may help optimize DBS outcomes.

The task of the neurosurgeon is to place the DBS electrode within the motor territory of the STN, and well within the STN borders such that current does not spread to the surrounding structures, thereby resulting in stimulation-limiting side effects (Richardson et al., 2009). Although the surgical procedure varies somewhat between medical centers, targeting of the STN during DBS surgery generally includes preoperative stereotactic imaging (MRI), used in conjunction with stereotactic atlases. This step is followed by intraoperative electrophysiological techniques consisting of the conversion of neural activity, in the form of single-unit neuronal activity (SUA) recorded at the microelectrode tip, into audio and visual signals. This procedure is experience-based and depends critically on the neurosurgeon's and neurophysiologist's ability to recognize entry into the STN, based on a variety of cues.

In order to obtain a three-dimensional map of the STN and surrounding structures, multiple microelectrode recording tracks (typically up to five) (Benabid et al., 2009) are carried out, either sequentially or simultaneously. Determination of the optimal track for DBS implantation is a key component to successful therapeutic outcome. Optimal track selection is primarily based on microelectrode recording of single unit activity (MER/SUA), which is used to identify cells with firing characteristics consistent with STN neurons and response characteristics confirming the motor sub-territory of the STN (Falkenberg et al., 2006). Despite the common usage of MER/SUA during stereotactic surgery for PD, limitations of this technique include difficulties interpreting complex signal patterns to localize the anatomical borders of the STN, highly overlapping spiking characteristics of single neurons around the target structure, recording SUA from a very small region, sensitivity of SUA to noise, susceptibility of SUA to small amounts of blood or edema within the microelectrode track, and the binary nature of SUA (unlike local field potentials; LFP), all of which may affect the accuracy of STN localization in PD (Chen et al., 2006; Gross et al., 2006; Novak et al., 2011). The caliber of single-unit recordings can be easily diminished due to drift of the recorded unit away from the electrode tip, as a consequence of transmitted pulsations of the brain and other environmental conditions (Sanghera and Grossman, 2004).

Interpretation of SUA recordings with computational intelligence was proposed as a new approach to help clinical decision making in the operating room (Wong et al., 2009). However, such approaches are still susceptible to the challenges of isolating single neurons in the operating room. LFPs represent the aggregate activity of neuronal populations, and are particularly sensitive to synchronous and oscillatory firing patterns (Priori et al., 2004; Gross et al., 2006). Recent studies indicate that LFPs in PD correlate with both motor and non-motor symptoms of the disease, and their signals are more robust than SUA (Priori et al., 2013; Thompson et al., 2014). Importantly, LFPs are an objective and quantitative metric while MER/SUA is more qualitative and subject to inter-practitioner variability. Although, the functional role of LFPs during DBS surgery is not fully established, we propose that they can be used to contribute to target localization in PD. In the present study, for the purpose of assisting with clinical decision making, we aimed to develop an automated approach by processing LFPs from multiple tracks to localize the dorsal border of STN and predict the macroelectrode implantation track identified by the neurosurgeon based on SUA interpretation. In the next sections first we describe our data collection methods and then detail our signal processing and classification techniques. We study the role of LFP sub-bands in prediction of the location of the dorsal border of STN. Moreover, we explore different decision criteria fused with LFP sub-band features toward prediction of optimal track selected by the neurosurgeons. We show experimental results obtained from 22 patients and discuss our results and demonstrate that LFPs can be used effectively in the operating room for clinical decision making.

## Methods

### Patients and surgical procedure

**Figure 1:**
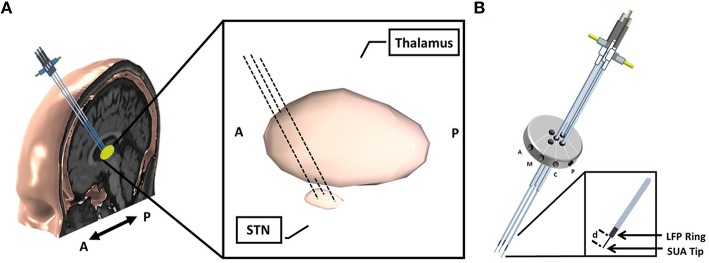
**Implantation of microelectrodes into STN using 3 simultaneous tracks. (A)** Schematic of 3-track- microelectrode implantation into STN. The schematic in the middle shows the 3D-structure of STN and thalamus. The anatomical structures are viewed from sagittal plane. **(B)** Schematic of 5-port Bengun routinely used in STN localization in PD and penetration of multiple microelectrodes through the Bengun. Among these three tracks, optimal track is used as the DBS electrode implantation track. The LFP recording surfaces of both AO recording microelectrode (NeuroProbe, AlphaOmega Inc., USA) and FHC microelectrode (MicroTargetingTM, FHC, Inc., USA) are identical with a 1 mm stainless steel semi-macro contact situated 3 and 1 mm proximal to the micro-recording tip (electrode offset “d”), respectively. A, anterior; M, medial; C, center; P, posterior. The fifth trajectory, not visualized in the figure, is lateral.

This is a multicenter study in which patients were recruited in either University of Minnesota Medical Center or Baylor St. Luke's Medical Center. The experimental protocol was approved by the Institutional Review Boards of the University of Minnesota and Baylor College of Medicine. All patients provided written informed consent to participate in the study. Intraoperative LFPs were recorded from 22 patients (14 men, 8 women), who were diagnosed with idiopathic PD, and exhibited typical motor symptoms which were tremor, rigidity, and bradykinesia. Disease duration ranged between 1 and 20 years, with a mean of 10.55 years (standard deviation of 4.7 years) (Table 1). All patients discontinued short-acting Parkinson's medications at 12 h prior to surgery, and long-acting medications at least 24 h prior to surgery. As per standard clinical protocol, target coordinates and trajectory to the STN, were identified by preoperative stereotactic MRI, which was fused to a stereotactic computed tomography (CT), on a neuro-navigational platform (StealthStation, Medtronic Corp, MN). Then, again based on standard clinical protocol, three simultaneous tracks were performed in each subject (Figure 1A). The superior and inferior borders of STN, along with the optimal depth for positioning the DBS electrode, were determined by the clinical team via electrophysiological mapping using MER/SUA, and the DBS electrode was implanted by the neurosurgeon based on these spatial data, followed by macro stimulation to confirm electrode location based on benefit and side effect profile—i.e., location within motor territory of STN, but not so close to border with adjacent internal capsule or medial lemniscus, that low-threshold stimulation- induced side effects were detected—followed by confirmatory intra-operative imaging modalities. Surgeries were performed in awake patients, under the benefits of local anesthesia. In 3 of 22 patients, microelectrode mapping of right and left STN occurred on different surgical dates, as the surgical procedures were staged for clinical reasons. Therefore, these recordings were counted as separate, enabling 25 individual STN microelectrode recordings for LFP-based optimal track prediction.

**Table 1:** **Clinical characteristics of the PD patients included in this study**.

**Number of patients**	**22**
Gender (women/men)	8/14
Age (mean ± std in years)	57 ± 11
Disease duration (mean ± std in years)	10.5 ± 4.7
**PHENOTYPES:**	
Typical	12
Tremor dominant	5
Bradykinetic/Rigid	5
OFF/ON UPDRS^†^ scores (mean)^‡^	45.60%
Number of microelectrodes recording (total)	75 in total

† UPDRS = Unified Parkinson's Disease Rating Scale.

‡ Pre-Operative Medication OFF-to-ON UPDRS Scores: Total Improvement.

### Intra-operative recordings and track selection

Following standard stereotactic techniques, and insertion of three brain cannulas and microelectrodes (Abosch et al., 2002), MER/SUA recording was carried out using a Microguide system (AlphaOmega Inc., USA) at 12 kHz. Simultaneous LFPs were recorded with an XLTEK-EMU128FS system (Natus, San Carlos, California) at 2 kHz with 16 bit A/D resolution or gHIAmp (gTec Inc., Graz, Austria), 38.4 kHz with 24 bit A/D resolution. The LFP recordings were obtained from a 1 mm wide stainless steel contact which is 3 mm (NeuroProbe, AlphaOmega Inc., USA) or 1 mm (MicroTargeting™, FHC Inc., USA) above the SUA recording tip and referenced to the cannula (Figure 1B). All microelectrodes were advanced toward the estimated target using a NeuroDrive (AlphaOmega Inc., USA) with micrometer resolution. In order to synchronize the SUA and LFP recordings, the digital depth information of the NeuroDrive is transmitted from the MicroGuide system to LFP recording system using a TCP/IP connection. Initial recordings began 20 mm above the intended final location of the electrode tip (“target”) as determined by direct targeting methods and proceeded until the electrode reached 3 mm below the MER-determined target, within the substantia nigra. Electrodes were lowered in 1 mm steps until 10 mm above “target,” and then in 0.5 mm steps. Duration of recordings at each depth was 15–30 s.

At each depth, the subjects sequentially rested and after a certain depth (< 10 mm) executed limb movements for 10–15 s period. The neurosurgeon used standard clinical techniques for localizing the STN, via real-time auditory and visual analysis of the recorded SUA. The dorsal, ventral, and posterior borders of the STN were identified by noting increased background noise and cell firing rate, and the STN neurons were examined for movement-responsive receptive fields. In particular, the superior border of STN was determined when the background activity increased and border cells were first observed among one of the tracks in MER-SUA. This position was used as the target value in STN border identification. Among three tracks, the track with the longest span of bursting cell firing and movement responsive fields was selected for the chronic DBS electrode implantation. This track was labeled as the “optimal track” and used as the target variable in LFP based track number prediction. The neurosurgeons were blinded to the LFP recordings in the operating room and the identification of STN dorsal border and the selection of optimal track for chronic DBS electrode implantation was not influenced by the LFP recordings. The STN border and the track selected by the neurosurgeon are predicted with the LFP data using the signal processing methods detailed below.

### Signal processing

All data were analyzed offline. Before any processing, all recorded signals were visualized with a custom in-house developed software and annotated to distinguish artifact and/or epochs of resting, active, and passive movements. Based on these annotations, resting state data were extracted into MATLAB (Mathworks, Natick, Massachusetts) and the data recorded by XLTEK system and gHIAmp system were downsampled to 1 and 1.2 kHz, respectively, for analysis.

**Figure 2:**
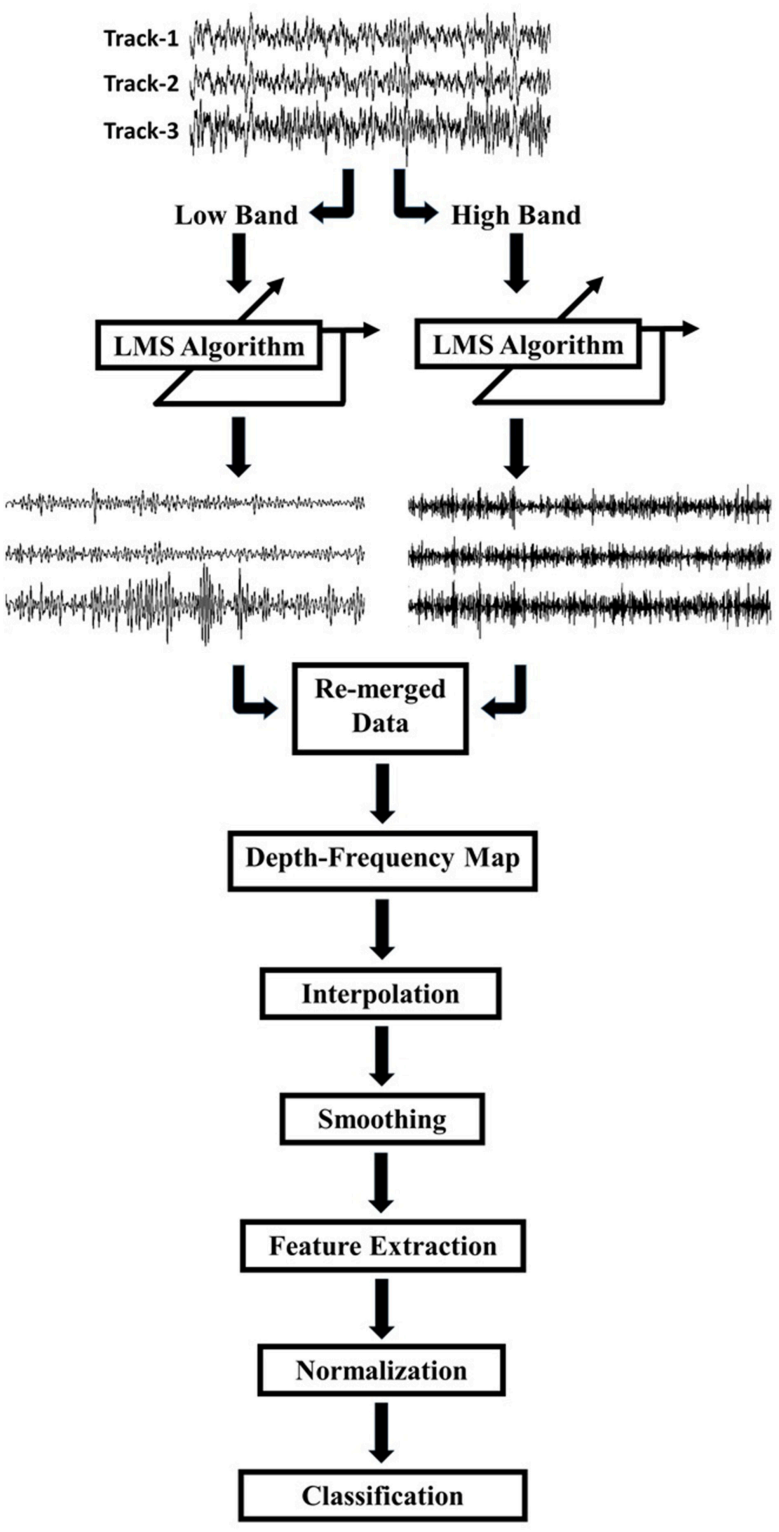
**Schematic of the work flow**. LFPs recorded by 3 tracks of microelectrodes were decomposed into low (8–200 Hz) and high (200–450 Hz) frequency bands. Each track was de-correlated using LMS algorithm at each frequency band. De-correlated signals were combined and the frequency domain features were extracted for classification.

A schematic diagram of our signal processing pipeline is given in Figure 2. As an initial step the raw signals were visualized and it was observed that tracks were difficult to distinguish, due to a high amount of common activity masking spatially localized activity and/or artifacts resulting from abrupt movements of the patient and other environmental factors. In order to eliminate the common activity among tracks, but still preserve the track-specific neural activity, the LFP data on each track were de-correlated using a least mean square (LMS) algorithm with a steepest descent update. The general formula for the de-correlation method is as follows:

(1) $$\begin{array}{r} \text{Initialization: }w\left(0\right)=0.5,\;n\;=0,1,2,\ldots \\ y\left(n\right)=\;w^{T}\left(n\right)x\left(n\right) \end{array}$$

(2) $$\begin{array}{r} e\left(n\right)=d\left(n\right)-\;y\left(n\right) \end{array}$$

(3) $$\hat{e}\left(n\right)=\{\begin{array}{ll} sign\left(e\left(n\right)\right)*\;20 & \text{if}\left|e\left(n\right)\right|>20\;\\ e\left(n\right) & \text{otherwise} \end{array}$$

(4) $$\begin{array}{r} w\left(n\;+\;1\right)=w\left(n\right)\;+\;\mu ê\left(n\right)x\left(n\right) \end{array}$$

where *y*(*n*) is the filter output, ê(*n*) is the residual which is the de-correlated signal, *d*(*n*) is the desired signal, μ is the step size, and *w*(*n*) is time varying filter coefficient (Hayes, 1996). In the current method, each channel, *d*(*n*), was predicted by using a linear weighted combination of other two channels, *x*(*n*). LFP activity from three tracks were recorded continuously during the entire surgery while the microelectrodes were traveling to the estimated target. Consequently, the signal characteristic varied over depths. Since in each depth the signal was recorded for 15–30 s, temporal variability exists in the signal. Therefore, the filter coefficients, w^*T*^(n), were updated on a sample by sample basis recursively to make the system to adapt to depth and time varying signal properties. At each iteration, the error, *e*(n), was calculated and this residual was used as the de-correlated LFP data in future steps for feature extraction and visualization. At 20 mm above the estimated target, all three tracks showed very similar signal characteristics indicating that they were in the white matter. Therefore, the initial filter coefficients were selected as the average of two channels with equal weights with the initialization of the filter coefficients *w*(*n*) = 0.5. By using this adaptive approach, we aimed to eliminate the common activity across tracks and suppress localized artifacts caused by patient movements and environmental factors. In order to prevent the system from being affected by high amplitude artifacts and to preserve the robustness, the error was saturated by using a 20 μV threshold (Equation 3). This threshold was determined experimentally and we observed that the system recovered from localized artifacts pretty fast even if the artifact amplitude was too large.

Due to differences in spatial correlation of low and high frequency bands, the monopolar data were, first, decomposed into two frequency bands which were 8–200 and 200–450 Hz by using a 2nd order Butterworth IIR filter (Figure 2). The LMS algorithm was individually applied to these subbands with step size of μ = 0.0002. Each track was de-correlated by using LFPs on the other two tracks. The algorithm was applied to each depth by transferring filter coefficients to the next depth. In this way, filter coefficients were not required to start from 0.5 at each new depth so that the algorithm would adapt faster and can use both temporal and spatial information of the past. Decomposed and de-correlated data were re-merged and spectral analysis was performed. In this regard, a modified Welch periodogram method with a robust statistics was used (Telkes et al., 2014). Specifically, a fast Fourier transform (FFT) was computed with a 1024 samples long Hanning window and the window was shifted with 50% overlap. Since some artifacts destroy or dominate the power spectrum estimate obtained with mean operator, the median of the spectra of all sliding windows was calculated to eliminate localized artifacts in the spectrum. The method was repeated for each depth and all spectra were combined to visualize depth-varying power spectrum of LFPs on multi tracks. Generated depth-frequency maps (DFMs) were resampled with a 0.25 mm depth resolution and linearly interpolated to obtain equidistance depth values. The maps were smoothed using a Gaussian kernel filter to suppress noise and to reveal beta and high frequency band oscillations (HFOs). Then, DFMs were normalized with the average baseline of three tracks and transformed into log scale using the Equations (5) and (6). The tracks were not normalized by their own baseline but by the mean of all three tracks in order to compare the signal power between them. The baseline used for normalization was selected as the highest depths which assumed to be in the white matter. Therefore, the baseline was determined as top 5 depths (20–15 mm above the estimated target) in 22 recordings. However, in rest of the three recordings, since the analysis started from lower than 20 mm (such that 18 mm) due to artifacts, the baseline segment was kept shorter and selected as top 3 depths. The purpose of using higher depths was to avoid from including any thalamic activity in normalization segment. The baseline normalization formula is noted below:

(5) $$\begin{array}{rcl} {\bar{b}}_{avg} & = & \frac{\left({\bar{b}}_{1}\;+\;{\bar{b}}_{2}\;+\;{\bar{b}}_{3}\right)}{3} \end{array}$$

(6) $$\begin{array}{rcl} {\bar{n}}_{dfm} & = & 20\;\times \;\log_{10}\left(\frac{{\bar{r}}_{dfm}}{{\bar{b}}_{avg}\;+\;\Phi \left(f\right)}\right)\; \end{array}$$

where ${\bar{b}}_{1},\;{\bar{b}}_{2},\;{\bar{b}}_{3}$ are the baseline spectrum of each 3 track, ${\bar{b}}_{avg}$ is the average baseline power, ${\bar{r}}_{dfm}$ indicates the DFM, Φ(*f*) is a small regularization parameter which is applied for each frequency *f* and ${\bar{n}}_{dfm}$ is the normalized DFM.

In order to observe the depth-varying frequency content of LFPs, DFMs of the patients were visualized. We noted that when the electrodes reached the STN border identified by the neurosurgeon, generally there was also an excessive activity in the beta and HFO range. In order to identify the most beneficial track along with the dorsal border of the STN, the sub-band power was extracted from all tracks and normalized by using a subject-specific average baseline. Based on the distribution of neural activity on the tracks, the sub-band frequencies were designated 11–32 Hz for beta band and 200–450 Hz for HFOs. The distribution of power in the STN among all tracks and the distribution of power only on the selected track inside and outside of STN (above the dorsal border of STN) were investigated by box and whisker plots. Student's *t*-test with two-sample was used to check if the distributions were significantly different or not.

### Classification

After sub-band power features were normalized between zero and one with a Max-Min normalization method for inter-subject comparison, a linear discriminant analysis (LDA) was used for classification. The principle of LDA is to maximize the separation of classes while keeping the within class densities small by using linear combination of features, $\vec{v}\cdot \vec{z}$ (Alpaydin, 2010). The linear discriminant function:

(7) $$\begin{array}{rcl} g_{i}\left(z|v_{i},\;v_{i0}\right) & = & v_{i}^{T}\;z\;+\;v_{i0}\\ = & \overset{d}{\underset{j=1}{\sum }}v_{ij}\;z_{j}\;+\;v_{i0} \end{array}$$

where *g*_*i*_(*z*) is the discriminant function for the input features *z*_*j*_ with sum of the weights *v*_*j*_ and threshold values *v*_*i*0_.

#### Localization of the dorsal border of STN

**Figure 3:**
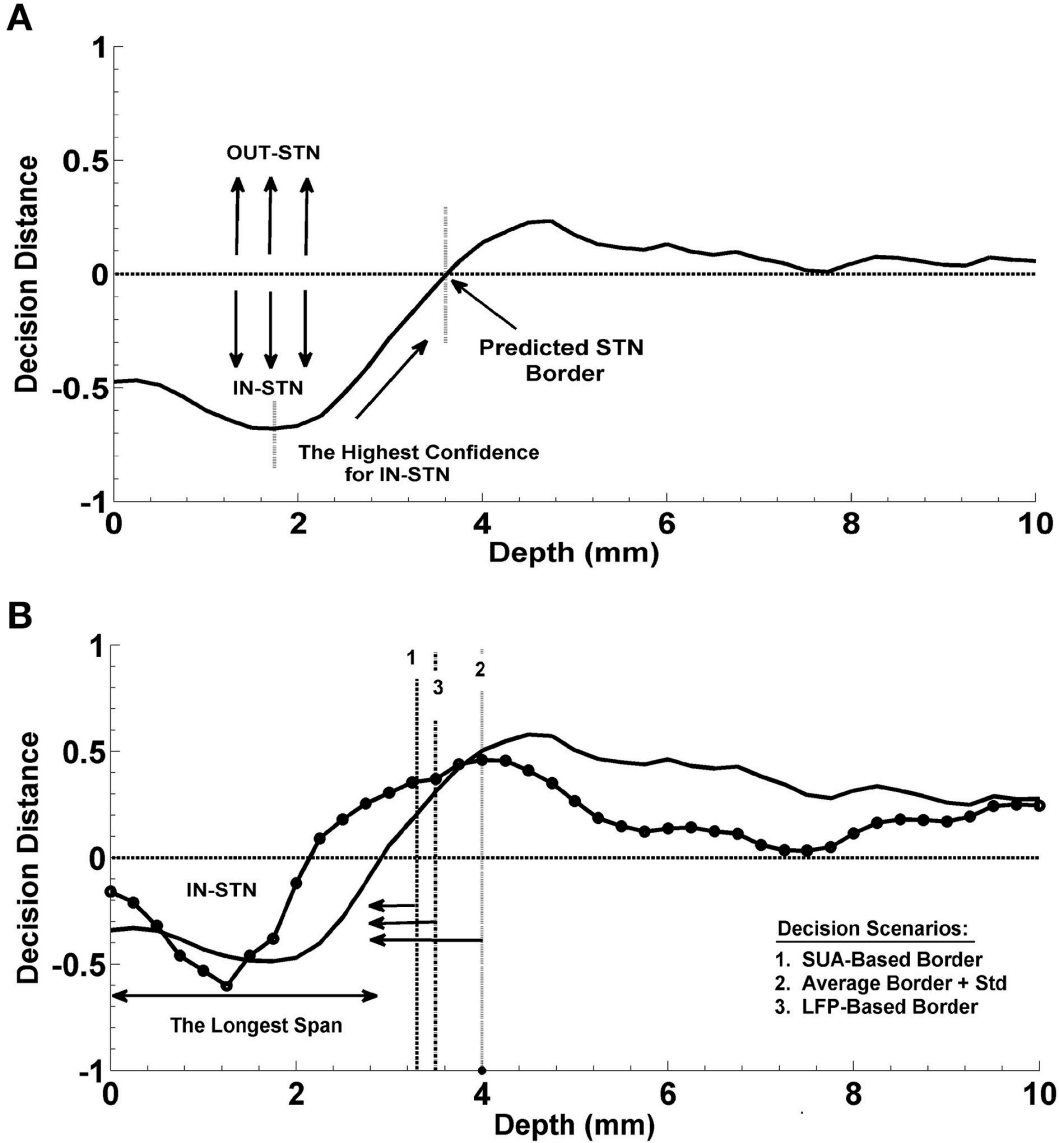
**Prediction of the dorsal border of STN and prediction of optimal track. (A)** Decision strategy in the prediction of dorsal border of STN. The classifier trained by contrasting the LFP sub-band features coming from inside and outside of STN gives a decision at each depth from top to bottom. These decision distances were used as a measure of confidence. Depth with the highest confidence for IN-STN decisions was marked. Then decisions were traced back where the LDA classifier voted for OUT-STN. This point where the classifier made IN vs. OUT decision transition was chosen as the predicted dorsal border of STN. **(B)** Decision strategy in the optimal track prediction. Classifier trained by contrasting the LFP subband features of selected track vs. un-selected tracks below the dorsal border of STN returned decision distances at all depths. The decision distances were used in three different scenarios: (1) Below the SUA-based STN border, (2) below the one standard deviation from the average of STN border, and (3) below the LFP-based STN border obtained from prediction of dorsal border of STN. The decision distance with the longest span was used as a decision criterion to predict the optimal track.

In the present study, the dorsal border of STN identified by clinical team is predicted from the depth varying LFP data by using the decision distance of a linear classifier as shown in Figure 3A. First an LDA classifier was trained by contrasting the LFP sub-band features coming from inside and outside of STN (above the dorsal border of STN). This classifier was evaluated at each depth and the returned decision distance was used as a measure of confidence. The depth with the highest confidence for IN-STN decisions was marked. Then we traced the decision distances above this depth and found the location where the LDA classifier voted for OUT-STN. This point where the classifier made IN vs. OUT decision transition was finally chosen as the predicted dorsal border of STN. The difference between prediction and the STN border identified by MER-SUA was calculated in each patient and the root mean square (RMS) of the prediction errors was used to quantify the performance of the classifier. Further, statistical analysis by using Student's *t*-test and *F-test* was conducted in order to compare the mean and the variance of predictions obtained by different subband features, respectively.

#### Prediction of the optimal track

The optimal track selection among three tracks is done by the neurosurgeons through interpreting the excessive single cell firings within the STN. Consequently, for the prediction of the optimal track using LFP data, an LDA classifier was trained by contrasting the LFP subband features of selected track vs. un-selected tracks below the dorsal border of STN. This classifier was evaluated at all depths as in the STN border prediction and the returned decision distance was used as a measure of confidence. The distance returned by the linear classifier was used in three different scenarios for final decision making (See Figure 3B). In the first scenario, the optimal track was predicted below the STN border provided by the neurosurgeon for that specific test subject based on the SUA interpretation. This represents the setup in which we fuse SUA- and LFP-based information. In the second scenario, no SUA information about the STN border of the test subject was used, and the decisions were given below the one standard deviation from the average of STN border estimated from all training subjects. In the third scenario, the optimal track decisions were made below the STN border which was derived solely from the LFP data. Specifically, here we explored whether or not the LFP could predict the optimal track without any SUA-based interpretation. We studied the classification performance below and above the STN border in these three different scenarios. A schematic diagram related to this process is given in Figure 3B. Depth-varying LFP subband features of each track were classified using the trained LDA and a label and related decision distance were generated by the classifier for each depth. We classified one of the tracks as the optimal one based on the longest span of decision distances voting for optimal track within the STN. Note that the longest span is a common approach used intraoperatively by neurosurgeons for MER-SUA-based optimal track selection. Also note that, if the track selected by neurosurgeon in the operating room did not match with the decision of the algorithm, the decision was counted as a misclassification.

The prediction of optimal trajectory was investigated using individual sub-band powers, beta and HFO, and their combination. To explore the benefit of the LMS algorithm over monopolar signals (raw signals), the same classification procedure was carried out with the raw data.

Finally, in order to assess the efficiency and reproducibility of the classification, a leave-one-subject-out method was used. In each step, one subject was used for testing, whereas the other subjects were used for training the LDA classifiers for STN dorsal border and optimal track prediction. The procedure was repeated until the whole sample was classified. In addition, this procedure was performed separately for individual beta and HFO sub-bands of LFP and their combinations to examine their efficacy in classification performance.

In order to explore a relationship between classification results and post-operative simulation parameters used for the initial programming 6 months after the surgery were compared in correctly classified and misclassified groups. The distribution of stimulation amplitude was investigated by box and whisker plots. Student's *t*-test with two-sample was used to check if the distribution of simulation amplitude, frequency, and pulse width were significantly different or not.

## Results

### De-correlation of LFP data from multiple tracks

**Figure 4:**
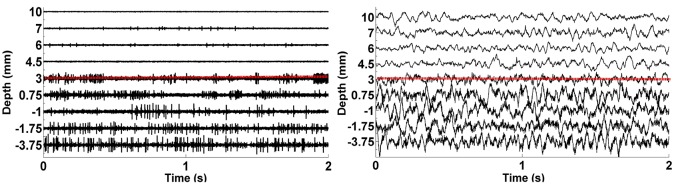
**The plots of raw SUA and LFP signals**. The graphic on the left shows the single neuron activity lasting 2 s while the graphic on the right indicates the aggregate activity of neuron populations at the same depths with the same duration. The dorsal border of STN shown as red dashed lines is 3 mm for this representative subject.

We analyzed LFP data derived from 75 MER tracks in patients with PD who were undergoing STN DBS electrode placement. Typical raw SUA and LFP data coming from various depths were shown in Figure 4. The red dashed line indicates the dorsal border of STN. The correlation between the two modalities is clearly seen. When the electrode enters the STN, both single cell firing and oscillatory activity increase.

**Figure 5:**
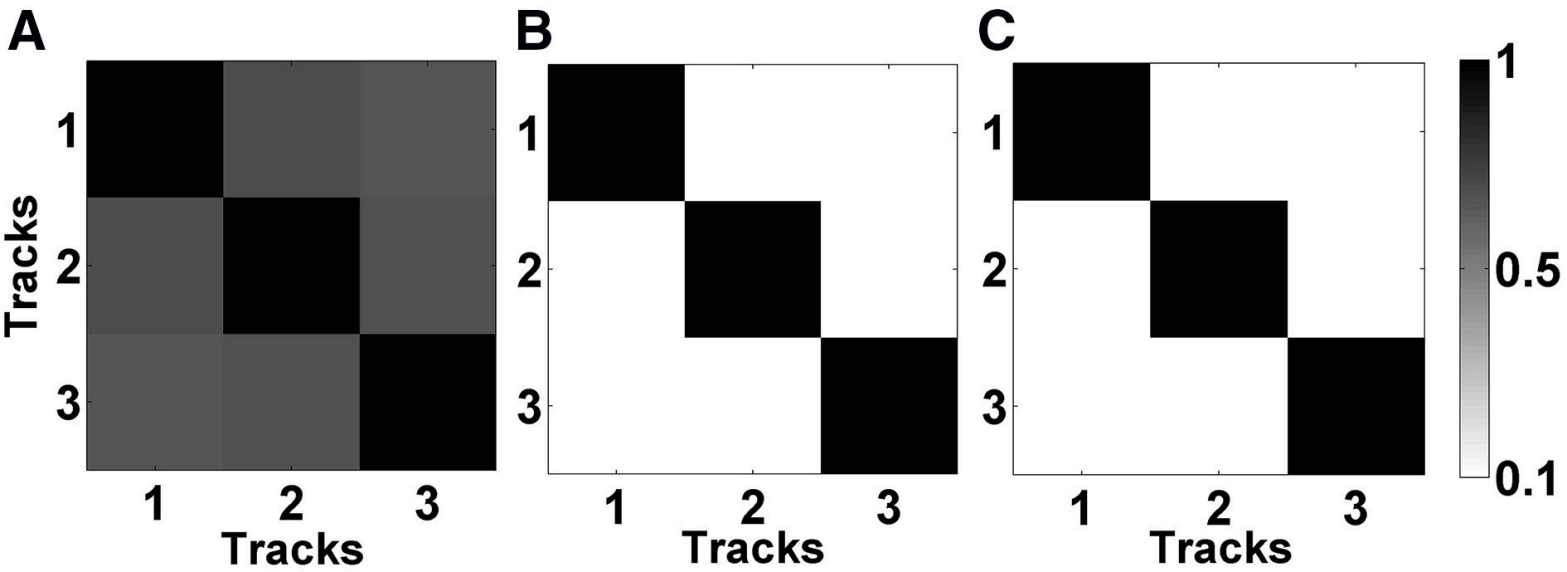
**Correlation matrices of raw and de-correlated data. (A)** The correlation matrix of raw data in beta band (11–32 Hz). All three tracks show high correlation. **(B)** The correlation matrix of raw data in HFO band (200–450 Hz). Small correlation between tracks indicate spatially more localized HFOs. **(C)** The correlation matrix of subband-decorrelated data. All three tracks demonstrate a small correlation indicating spatio-spectrally distinguished LFPs.

In Figures 5A,B, the mean correlation matrices of raw LFP data filtered in beta band (11–32 Hz) and HFO band (200–450 Hz) were shown. The correlation matrix in the beta band (Figure 5A) explicitly shows that the spatial correlation between tracks is high whereas the correlation between tracks in HFO range is small (Figure 5B). The small amount of correlation at HFO band in raw data also shows that oscillations at higher frequencies are more localized than the oscillations at lower frequencies. For these reasons, the LFP data were de-correlated with LMS separately in these frequency bands. It was found that the correlation between tracks is reduced after the LMS-based preprocessing step (Figure 5C) which helped more to distinguish the tracks.

**Figure 6:**
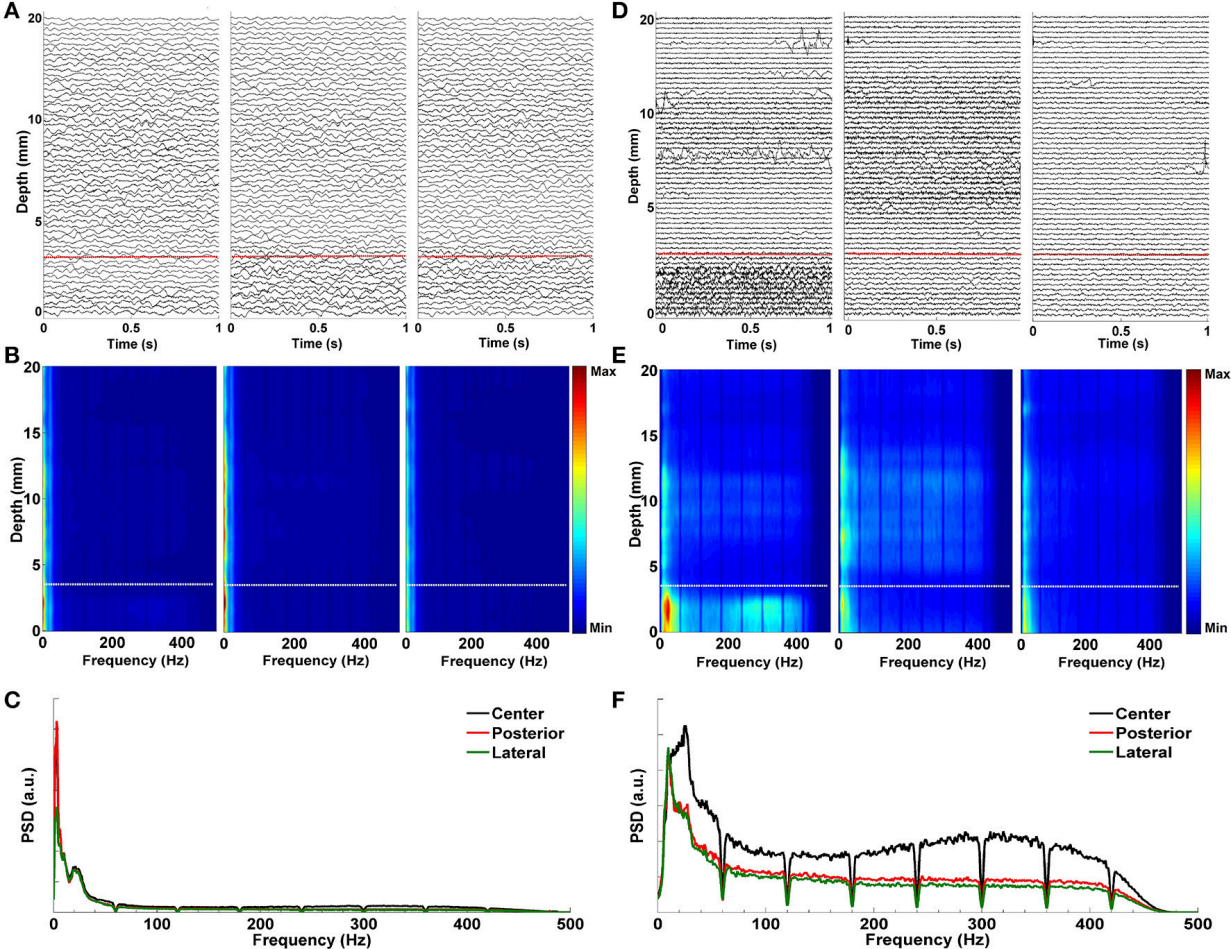
**Effect of LMS algorithm by comparing it to the raw LFP signals. (A)** Raw LFP distribution. Raw LFPs were recorded from 20 mm above the estimated target down to 0 mm. Distribution of raw LFPs in each depth shows no visible difference between tracks. Red dashed line indicates the dorsal border of STN. **(B)** Depth frequency map (DFM) generated from raw LFPs. Neither pathological beta oscillations nor HFOs are visible in tracks. The high-energy and low band common activity masks other oscillations. White dashed line indicates the dorsal border of STN. **(C)** Power spectrum of raw LFPs. It was generated from the LFP data below the dorsal border of STN. The high correlation between tracks in low frequencies can be clearly seen. Since HFOs are masked by these low frequency oscillations, it is not possible to distinguish them in the spectrum. **(D)** De-correlated LFP distribution. Effect of LMS algorithm on the raw LFPs is clearly seen. Target specific oscillations are visible on de-correlated LFP data. The red dashed line shows the dorsal border of STN determined by MER-SUA. **(E)** DFM of de-correlated LFPs in each microelectrode track. The high energy oscillations in distinct low and high frequency bands inside the STN (below the white dashed line) can be clearly seen in the first track. **(F)** Power spectrum of de-correlated LFPs. Tracks do not show excessive and correlated beta activity anymore. There is an increase in the gamma band (35–55 Hz) and great enhancement in HFO range (200–400 Hz) in the first track compared to other tracks.

Figure 6 demonstrates the effect of LMS algorithm by comparing it to the raw LFP signals. As it can be seen in Figures 6A–C, the raw LFP data have a high amount of common activity across all tracks at various depths which masks the spatially and temporally distinguishing patterns during targeting. In Figure 6B, DFMs indicate that the high-energy low band activity among tracks masks other oscillations. The common activity across three tracks and the high energy low band oscillations can be also seen in the power spectrum shown in Figure 6C which was generated from the LFP data below the dorsal border of STN. On the other hand, target specific oscillations are clearly seen on de-correlated LFP data (Figure 6D). In particular, the energy in the first track is much higher than the other two tracks and it is easier to observe the track differences and the estimated STN border depth for the target localization. The DFMs of these tracks shown in Figure 6E demonstrate that the first track contains LFPs with higher energy in low and high frequency bands below the dorsal border of the STN which is marked with a white dashed line. Furthermore, the power spectrum shown in Figure 6F demonstrates that not all three tracks show excessive beta activity. There is an increase in the gamma band (35–55 Hz) and great enhancement in HFO range (200–400 Hz) in the first track compared to other tracks. The LMS algorithm not only reveals the pathological beta oscillations but also the HFOs having lower energy.

### Spatio-spectral patterns of multitrack LFP

**Figure 7:**
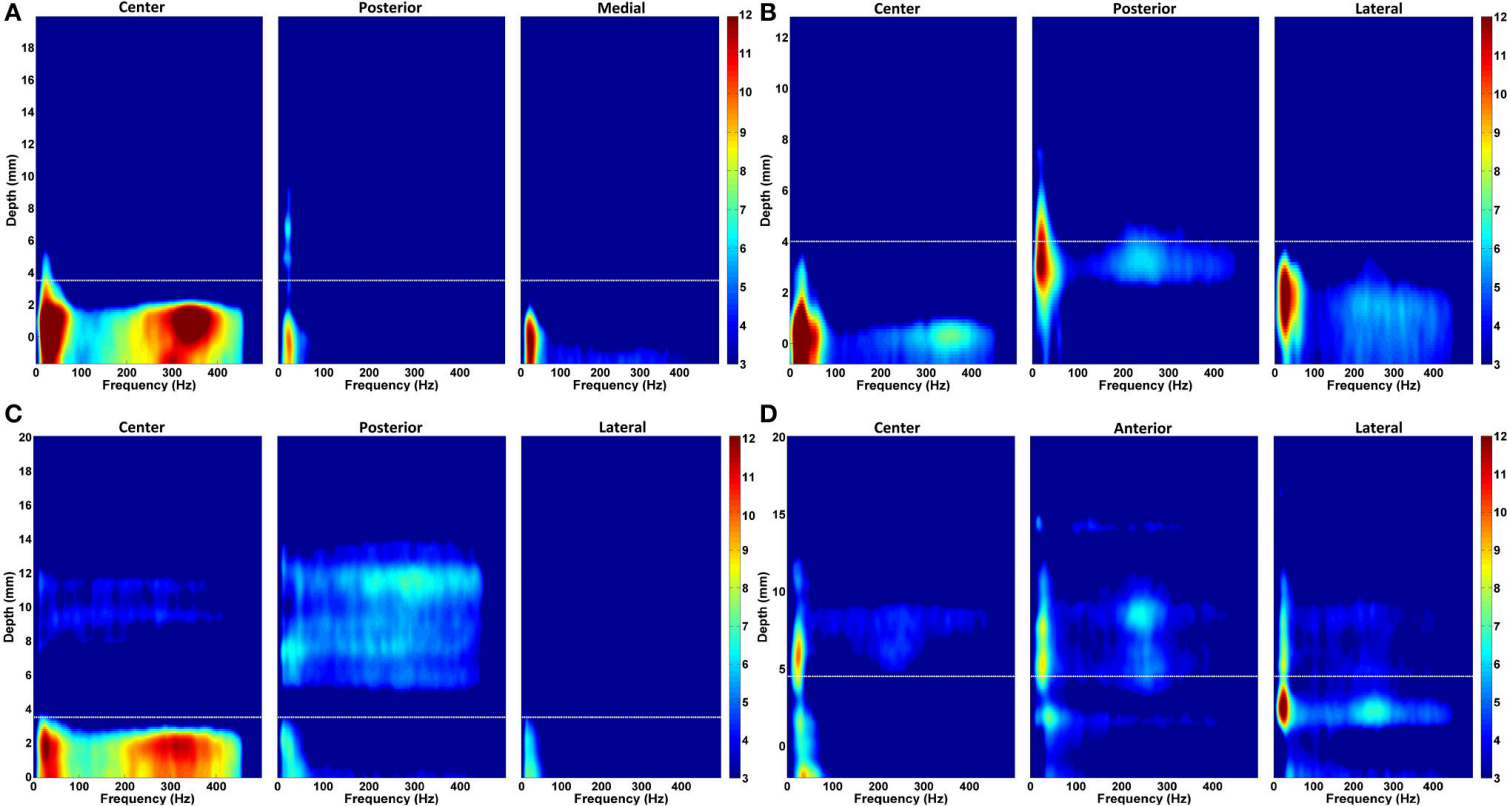
**Normalized depth-frequency maps**. Representative normalized DFMs of de-correlated LFP data of all three tracks from four representative patients are shown. **(A)** The excessive beta oscillations can be clearly seen in the first subject dominantly in the center track and localized to certain depths. There is a strong and track-specific HFO around 350 Hz which is well aligned with the low band activity. In this subject, selected track by neurosurgeon for the DBS electrode implantation is center track. **(B)** Beta oscillations are observed in all tracks along with HFOs. The HFO in the center track sits at 350 Hz while it is located at 250 Hz in the posterior track. The lateral track shows wider but weaker oscillations. The selected track by neurosurgeon is lateral. **(C)** A similar LFP characteristics to the first subject (shown in **A**) with dominant beta oscillations and HFOs in the center track can be seen. Similarly, these oscillations are well aligned below the border and highly stronger than the beta oscillations in the other two tracks. Center track is selected for DBS electrode implantation. **(D)** These DFMs show different LFP characteristic compared to others. None of the tracks shows strong, long span beta oscillations. Specified border is not aligned with beta oscillations. All three tracks demonstrate spatially different weak-to-minor HFOs. The white dashed line indicates the dorsal STN border. In this subject, the selected track is anterior.

In order to provide a sense of the depth-varying frequency content of multitrack LFPs, we demonstrated representative normalized DFMs of de-correlated LFP data of all three tracks from four patients in Figure 7. In each map, the dorsal STN border is marked with a white dashed line. The excessive beta oscillations can be clearly seen in the first subject dominantly in the center track and localized to certain depths (Figure 7A). The power of beta oscillations in the posterior and medial track is weak, yet it can be still observed. Furthermore, there is a strong and track-specific HFO around 350 Hz which is well aligned with the low band activity. On the other hand, in the subject presented in Figure 7B, beta oscillations are observed in all tracks along with HFOs. Although the excessive LFP activity occurs below the STN dorsal border as for the patient presented in the Figure 7A, the excessive depth varying spectral patterns are pretty track and region specific. The HFO in the center track sits at 350 Hz while it is located at 250 Hz in the posterior track. The lateral track shows wider but weaker oscillations. DFMs in Figure 7C demonstrate similar LFP characteristics to the first subject (Figure 7A) with dominant beta oscillations and HFOs in the center track. Similarly, these oscillations are well aligned below the dorsal border and highly stronger than the beta oscillations in the other two tracks. Distinctly, strong oscillations at higher depths are observed above the dorsal border in the posterior track which might be related to thalamic activity. Note that we observed high frequency activity localized at higher depths above the dorsal border of STN in at least one of the un-selected tracks in 56% of recordings. The number of the un-selected tracks with the observed oscillations were: 8 posterior, 5 center, and 1 medial. Similar to the HFO activity seen in Figure 7C, these oscillations were noted from 11.5 ± 2.6 to 5.7 ± 2.4 mm (average values) above the estimated final location of the electrode tip. The tracks having higher-depth HFOs do not include strong beta activity. These oscillations have a longer spatial span with lower power. It is likely that these oscillations rise from thalamic structures (Hutchison et al., 1998; Falkenberg et al., 2006), and given their spatial distribution in relation to the beta band activity, they might be used as markers for STN localization. The fourth representative subject shown in Figure 7D introduces a different LFP characteristic compared to others. None of the tracks are associated with strong, long span of beta oscillations. Specified border is not aligned with weak beta oscillations but the short lasting excessive one in the lateral track. All tracks demonstrate spatially different weak-to-minor HFOs. The overlap in the LFP activity between tracks, the weak activity across tracks and thalamic oscillations are some of the factors contributing to the challenges to the prediction of dorsal border of STN and selection of optimal track.

#### Intra-track and inter-track differences of LFP spectra

**Figure 8:**
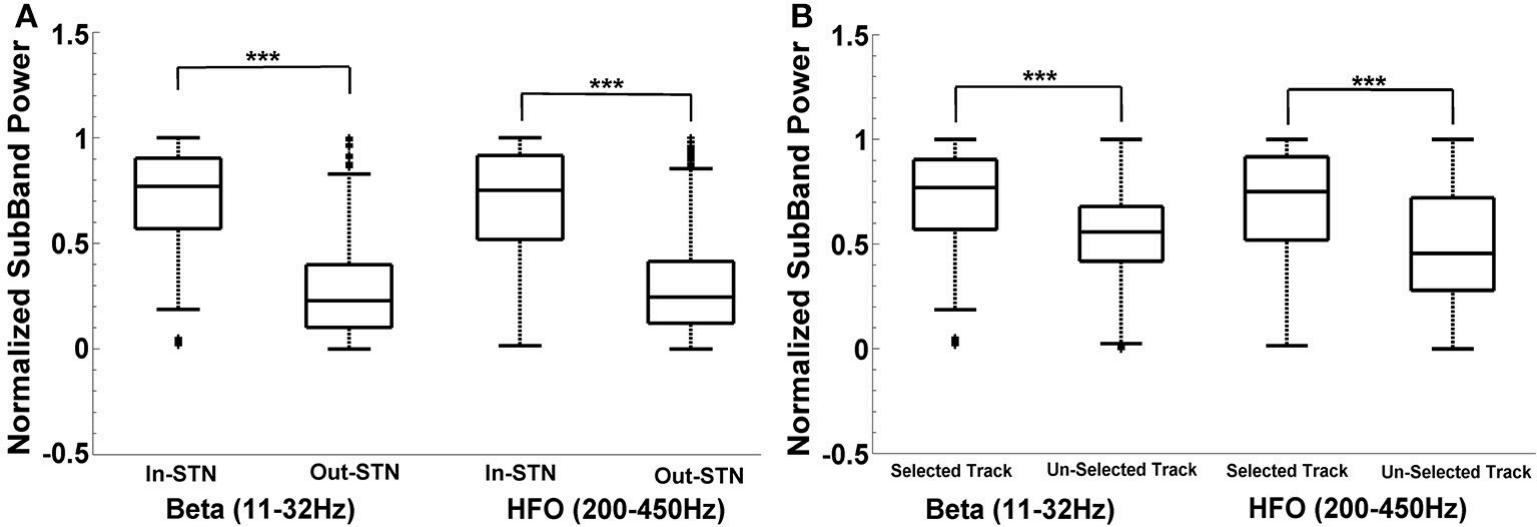
**Intra-track and inter-track differences of LFP spectra**. Box plots demonstrate the normalized subband power of all subjects. Panel **(A)** shows the IN and OUT of STN power distribution in the selected track in beta (11–32 Hz) and HFO (200–450 Hz) bands. The sub-band power is much higher inside the STN. The difference between the power inside and outside the STN region (above the STN dorsal border) is statistically significant in each specified frequency band (*p* < 0.001). Panel **(B)** shows the IN-STN power in the selected vs. un-selected tracks. Both distributions are statistically significant in each frequency bands. ^***^Statistically significant difference between distributions (α = 0.01).

For the neurosurgeon selected track, the distribution of beta and HFO subband powers above and below the dorsal border of STN are given in Figure 8A. The analysis shows that there is a significant difference between the power inside and outside the STN region (above the STN dorsal border) (*t* = 44.72, *p* < 0.001; *t* = 34.89, *p* < 0.001) in the selected track. As seen from the box-plot in Figure 8A, the sub-band power is much higher inside the STN. When the subband power was compared between the optimal and other tracks (Figure 8B), the distributions were found significant as well (*t* = 16.47, *p* < 0.001; *t* = 15.17, *p* < 0.001). The significance is consistent at the beta band and HFO band in both distributions. The variance of HFO power in the un-selected tracks is higher than the variance in the selected track. Based on our previously mentioned findings, we postulate that thalamic activity in un-selected tracks might contribute to increased variance of HFO power when the distribution includes entire track.

### Localization of the dorsal border of STN

**Figure 9:**
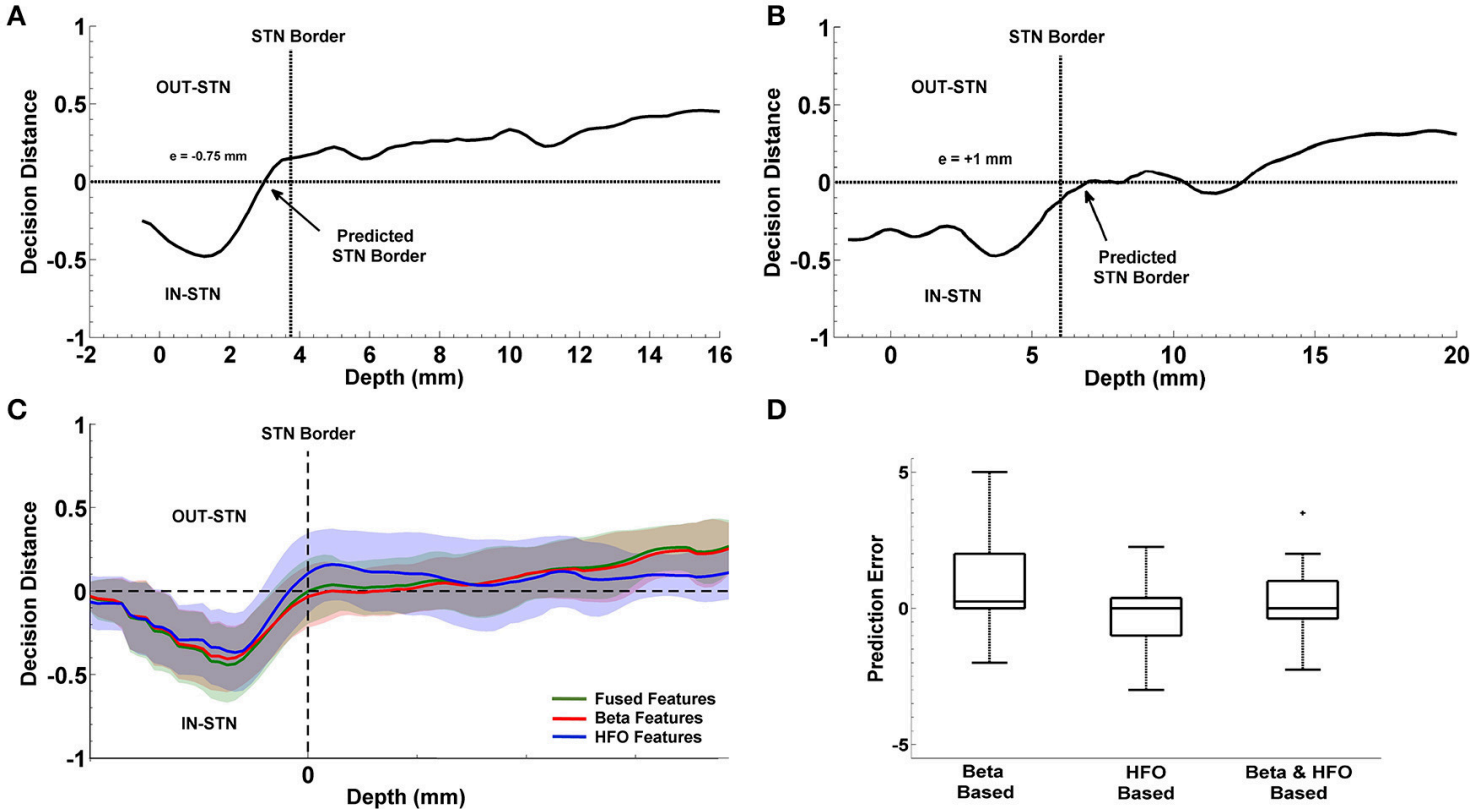
**Localization of dorsal border of STN**. Panels **(A,B)** show the decision distances returned by the classifier voting either for IN-STN or OUT-STN for two representative subjects. Predicted STN border is shown with an arrow corresponds to the position where we find the maximum confidence point voted for IN-STN and trace back to the depth crossing zero. The black dashed lines indicate the dorsal border of STN obtained by MER/SUA. The predicted border is 0.75 mm lower than the SUA-based border in the first subject **(A)** and 1 mm higher in the second **(B)**. Panel **(C)** demonstrates the average border decisions with the standard deviation coming from all test subjects by using individual sub-band powers, beta (red) and HFO (blue), and fused features (green). **(D)** Comparison of prediction errors. The error (RMS) of the predictions obtained with beta and HFO band features are 1.98 and 1.18 mm. The RMS of the error in predictions obtained from fused features is 1.22 mm. The variance of RMS values were compared by *F-test*. The difference between beta-based and HFO-based prediction error is marginally significant (*p* = 0.054) with α = 0.05. ^+^indicates the outlier error value.

The progression of prediction of STN dorsal border for representative subjects and the average results estimated from the entire patient population is shown in Figure 9. In Figures 9A,B, decision distances returned by the classifier voting either for IN-STN or OUT-STN are shown for two representative subjects. The decision distance curves were obtained from the fused beta and HFO features. Note that the predicted STN border is shown with an arrow corresponds to the position where we find the maximum confidence point associated with IN-STN and trace back to the depth crossing zero. The dorsal border of STN provided by the neurosurgeon based on SUA interpretation is shown with a dashed vertical line. Figure 9A shows a late prediction of the dorsal border (*e* = −0.75 mm) while Figure 9B indicates an early border prediction (*e* = +1 mm). Figure 9C demonstrates the average border decisions with the standard deviation coming from all test subjects by using individual sub-band powers, beta and HFO, and fused features. The overall localization error of the dorsal border of STN was quantified by calculating RMS of the error between target values and LFP predictions across all subjects. The red and blue lines show the decisions obtained with beta and HFO band features indicating an RMS error of 1.98 mm and 1.18 mm, respectively. The mean value of prediction error for beta band features was 0.83 ± 1.84 mm while the mean of error for HFO band features was −0.23 ± 1.18 mm. The decisions obtained through the fused beta and HFO band features had an RMS error of 1.22 mm with mean of 0.24 ± 1.22 mm. In Figure 9D, the distribution of prediction errors are shown for each studied subband and their fusion. Student's two sample *t*-test analysis indicated that the difference between mean values of beta-based prediction error and HFO-based prediction error was significantly different (*t* = 2.22, *p* = 0.0322) while no statistically significant difference was found neither between beta-based prediction error and the error of fused features (*t* = 1.23, *p* = 0.2244) nor HFO-based prediction error and the error of fused features (*t* = −1.25, *p* = 0.2185). When the variances of these distributions were compared by using an *F-test*, the analysis showed that the difference between beta-based and HFO-based border prediction was only marginally significant [*F*_(1, 2)_ = 2.42, *p* = 0.054] while there was no statistically significant difference between the variance of individual sub-band powers (beta and HFO) and fused power [*F*_(1, 3)_ = 2.26, *p* = 0.075; *F*_(2, 3)_ = 0.94, *p* = 0.88, respectively].

### Prediction of the optimal track

We studied the optimal track classification in three different scenarios using the LFP data: (i) below the SUA-based STN border, (ii) below one standard deviation from the average STN border obtained from training data, and (iii) the LFP-based STN border. We trained the LDA classifier using individual subband powers and their combination. Our results toward the prediction of optimal track from LFP data is given in Table 2. We note that the best results were obtained from the combined subband power features and consistently in all these scenarios, the optimal track prediction accuracy was 80% (shown in bold type) indicating that the classifier can predict the track targeted to the STN in 20/25 recordings. These results show that prediction of optimal track can be performed independently from single unit recordings. When the beta and HFO subband features were used individually, the classification accuracy dropped to 72 and 68% respectively. When the procedure was repeated with the raw data, the prediction rate was poor. In particular, the classification accuracy was 64% for beta band power and 68% for the HFO and fused features which supports the observation that HFOs obtained in monopolar configuration are already highly de-correlated among different tracks.

**Table 2:** **Prediction rates of classifier**.

**Power of IN STN in all tracks**		
	**LMS data**	**Raw data**
Beta	0.72	0.64
HFO	0.68	0.68
Beta & HFO	**0.80**	0.68

Despite the spatially localized thalamic oscillations, the classification results obtained above the STN border were quite poor. The prediction accuracy was found to be 40% when the classification was computed above SUA-based or LFP-based STN border. Decision accuracy with average STN border was even lower at 36% by using fused sub-band power. The results indicate that the LDA classifier trained with the LFP features above the STN does cannot predict the optimal track with a reasonable accuracy and was close to chance level.

**Figure 10:**
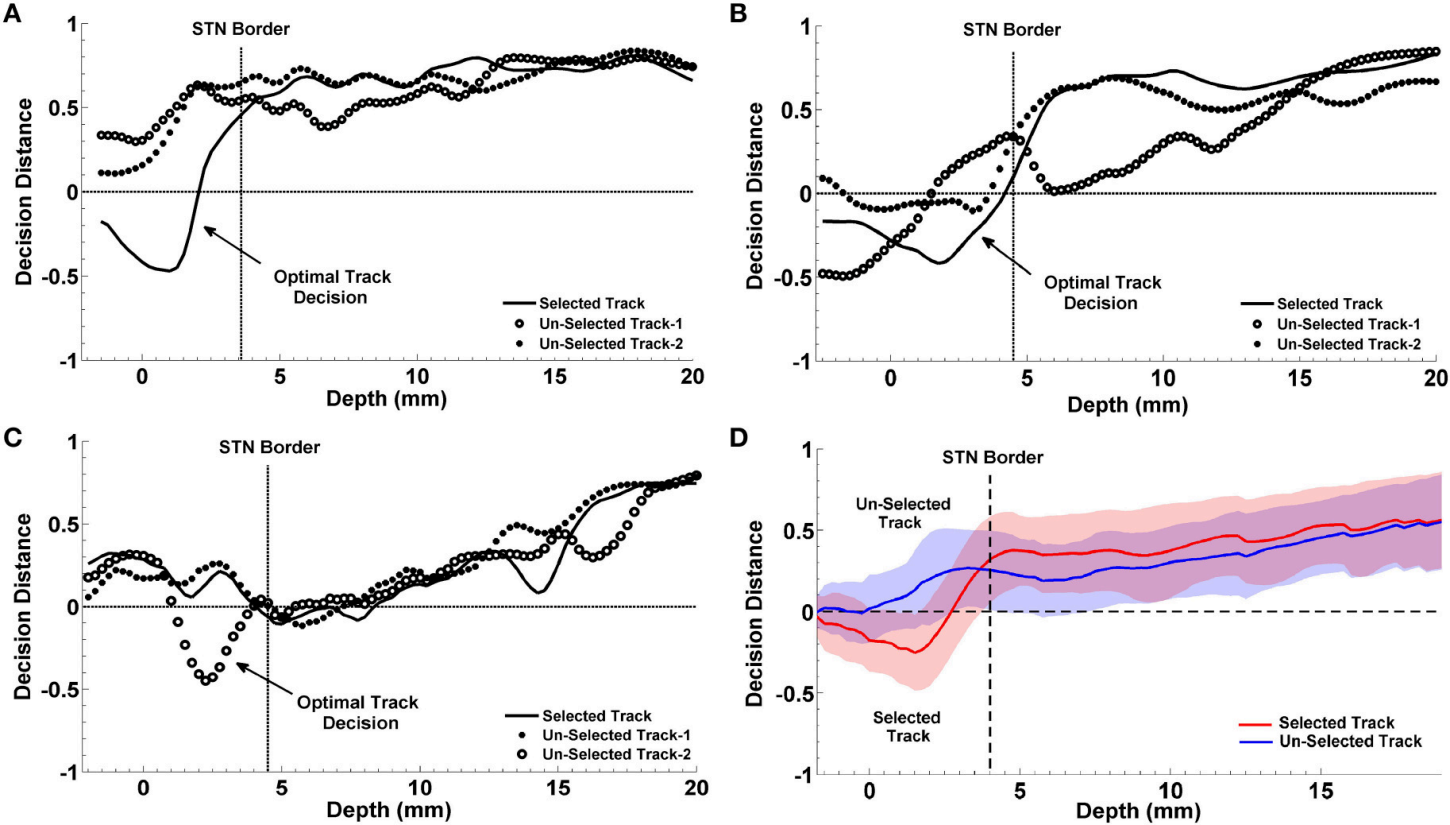
**Progression of classification in optimal track prediction**. The progression of classification over depths for three representative subjects are shown in **(A–C)**. The black dashed lines show the STN border provided by the neurosurgeon based on the SUA interpretation. The decision distances with the highest confidence and the longest span below the STN border indicate the optimal track. Panel **(A)** shows that decision distance is reaching the highest confidence level only in selected track which is determined as the optimal track by the algorithm. Panel **(B)** shows that all tracks pass the zero line however, only the selected track points the longest span of decisions. So, the selected track is determined as the optimal track by the classifier. Panel **(C)** demonstrates a misclassified subject. Since only the un-selected track-2 is passing zero line and reaching the highest confidence level, it is determined as the optimal track by the algorithm. Panel **(D)** shows the average optimal track decisions with the standard deviation coming from all test subjects. It shows a clear separation in decision distances between selected (red) and un-selected tracks (blue) indicating a high percentage of correct prediction among the subjects.

The progression of classification over depths for three representative subjects are shown in Figures 10A–C. In each plot, the STN border location provided by the neurosurgeon based on the SUA interpretation is also represented with a vertical dashed line. The decision distances in both selected and un-selected tracks returned by the classifier are close to each other down to the dorsal border of STN. Since the spectral characteristics of LFPs change inside the STN compared to higher depths, we observe a sudden change between the decision distances as well. If only one of the tracks deviates from the others and reaching the highest confidence level, it is easily determined as the optimal track by the classifier. If more than one track are voted for the optimal track (below the zero line in Figure 10) with high confidence levels, algorithm gives the optimal track decision by computing the longest span of the selected track votes. The progression of the classification for a misclassified subject is given in Figure 10C. The average decisions for the optimal track of all subjects with the associated standard deviation are given in Figure 10D. A clear separation is observed in decision distances between selected and un-selected tracks indicating a high percentage of correct prediction among the subjects.

#### Distribution of selected tracks

Table 3 shows the frequency of selected tracks based on MER-SUA interpretation and LFP processing. As per standard clinical protocol, the initial trajectory to target STN is determined by preoperative stereotactic imaging. Three tracks are selected by the neurosurgeon based on the initial planning for microelectrode recordings. The initial expectation is that the center track will hit the STN while other tracks account for possible targeting error. Then based on the MER-SUA recordings the optimal track is selected among these three trajectories. Although the image based planning aims to hit the STN with the center track, Table 3 demonstrates that intraoperative MER-SUA-based decisions among 25 recordings is not biased toward the center track. We note that the selection frequency is higher in anterior track based on MER-SUA mapping. In addition, the posterior track is not selected at all. When the selection frequency based on LFPs is studied, it can be seen that both MER-SUA and LFP decisions (shown in bold) match with a high percentage but LFP based prediction was more in favor of the center track. Overall, our results indicate that stereotactic planning does not perfectly correlate with intraoperative electrophysiology based track selection and highlight the variance in track selection.

**Table 3:** **Frequency of track selection in the present study**.

**Comparison of SUA-based and LFP-based track selection**		
	Anterior: 10/**9**	
Medial: 2/**1**	Center: 8/**10**	Lateral: 5/**4**
	Posterior: **0/1**	

The selection frequency based on LFPs are shown in bold type.

#### Post-operative programming parameters

**Figure 11:**
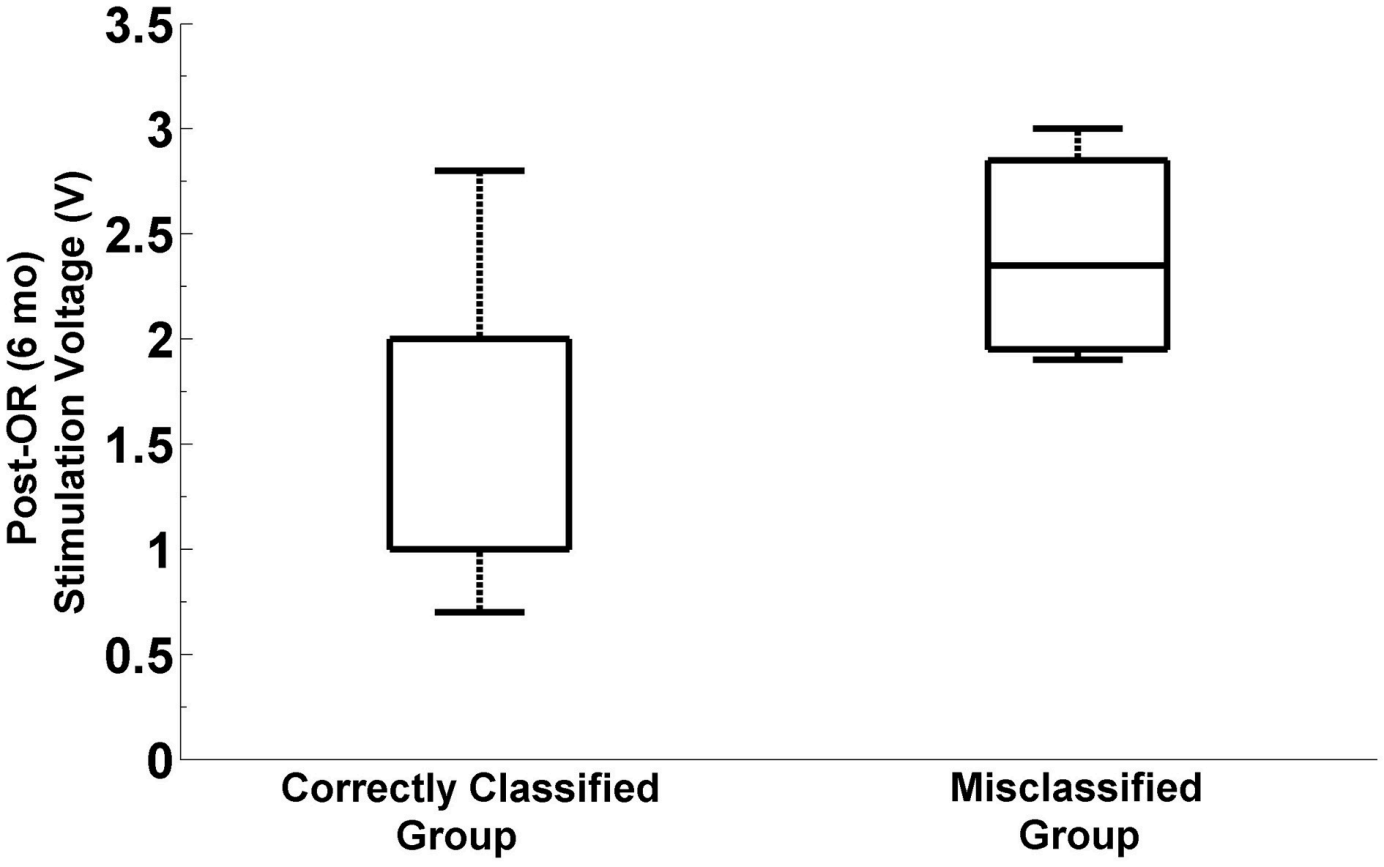
**Distribution of post-operative stimulation voltages in correctly classified and misclassified groups**. Box plots show the stimulation voltages which were used in two groups during the programming 6 months after the surgery. The box plot on the right indicates the distribution of post-operative stimulation voltages in misclassified group by excluding the outlier subject with 1V stimulation. The difference between stimulation voltages in the correctly classified and misclassified group is marginally significant (*p* = 0.068, α = 0.05).

We explored whether there exist any difference in programming parameters between the correctly classified and misclassified patients. In particular we investigated the post-operative simulation parameters such as voltage, frequency and pulse width which were selected during the programming 6 months after the surgery. The distribution of selected stimulation voltages are presented in Figure 11. We note that the average stimulation voltage used in correctly classified group is 1.72 ± 0.63 V while it is 2.12 ± 0.69 V in misclassified group. Student's two sample *t*-test analysis indicates that the difference in voltages between two groups is not statistically significant (*t* = −1.16, *p* = 0.2595). However, one of the misclassified subjects we observed has high level beta and HFO activity in both selected and un-selected track and this patient is stimulated with 1 V. This indicates that both tracks could be viable. When this subject is removed from the misclassified group, we note that the mean post-operative stimulation voltage increases to 2.4 V ± −0.46 for the misclassified population. The difference in post-operative stimulation voltages between correctly classified and misclassified groups without this outlier becomes marginally significant (*t* = −1.92 *p* = 0.0685). No significant difference is found either in the frequency (183.1 ± 5.8 Hz) or in the pulse width (90 ± 26.5 μs) between groups (*t* = −0.74, *p* = 0.4692 and *t* = 0.96, *p* = 0.3477, respectively).

## Discussion

Significant variability exists in the axial and coronal orientation of the STN in humans (Patel et al., 2008), and the motor territory of the STN is small, measuring ~4–6 mm extent from dorsal to ventral. These factors combined with brain shift between preoperative stereotactic imaging and intraoperative electrode brain penetration can lead to targeting errors in the operating room. Pre- and intra-operative clinical imaging methods alone are suboptimal for accurate placement of a DBS electrode; they are subject to distortion, and visualization of a clear differentiation between the STN and surrounding structures can be difficult. In this regard, our study also indicates a considerable amount of variance in track selection. Under the assumption of hitting the STN through center track by image based planning, track selection was not found to be biased toward the center track in the intraoperative MER/SUA-based decisions indicating that stereotactic planning does not perfectly correlate with intraoperative electrophysiology based track selection.

Accurate localization of STN motor territory through intraoperative electrophysiology is a crucial step for DBS electrode implantation (Zonenshayn et al., 2000; Gross et al., 2006). As recently as 2013, an international survey of high-volume DBS implanting sites revealed that 83% of centers use microelectrode recording indicating that the most commonly used electrophysiological mapping method remains MER-SUA recordings (Abosch et al., 2013). However, the method has several limitations in practice as subjective interpretation of complex signal patterns to localize the anatomical borders of the STN, being less stable and more “susceptible to technical (e.g., impedance) and physiological (e.g., cerebrospinal fluid and blood) fluctuations” (Thompson et al., 2014). As Gross et al. indicated, the number of groups using solely macroelectrode/DBS mapping to target the STN without any microelectrode recording is high (Chen et al., 2006; Gross et al., 2006; Telkes et al., 2014). Although there are advantages of using macroelectrode/DBS technique alone in STN targeting like carrying less amount of risk for intracranial hemorrhage since there is no multiple trajectories and due to the blunt-tip of the macroelectrode (Xiaowu et al., 2010), the drawbacks like microlesion effect which might limit the clinician's ability to test or therapeutic effectiveness in the operating room and poor spatial resolution should be taken into consideration (Rezai et al., 2006; Wang et al., 2014). Although asleep, MRI-based non-MER-guided surgery is gaining sway (Starr et al., 2014), the possibility of clinically testing a DBS electrode prior to permanent implantation does not exist as yet in the context of such a procedure.

Earlier investigations have documented that excessive beta oscillations in certain basal ganglia structures, especially the STN, represent a pathophysiological feature of PD (Weinberger et al., 2006; Kane et al., 2009; Lopez-Azcarate et al., 2010; Oswal et al., 2013). Excessive beta band (8–30 Hz) activity is detected when the electrodes enter into the STN (Levy et al., 2002; Kühn et al., 2008; Brittain and Brown, 2014). Similarly, excessive oscillations at very high frequency ranging from 200–400 Hz are also observed (Priori et al., 2004; Lopez-Azcarate et al., 2010; Özkurt et al., 2011). Even though these high frequency oscillations are considered to represent a pro-kinetic state, and appear with dopaminergic medication and/or induced movement (Foffani et al., 2003, 2006; Trottenberg et al., 2006), others have demonstrated that HFOs (>200 Hz) can still be observed in the STN during the medication OFF state or at rest (Lopez-Azcarate et al., 2010). In our study, all patients discontinued with their short and long acting medication before the surgery and were in OFF state. As others, we observed HFOs in the resting state and increased band power along with entry into the STN. Existence of excessive beta band and HFOs within the STN in PD can be used in target localization. However, the variability and patient-specific characteristics of spatial distribution of excessive beta and HFOs should be taken into consideration (Chen et al., 2006; Weinberger et al., 2006; Wang et al., 2014).

Despite a few publications using intraoperative microelectrode LFPs for STN localization (Michmizos et al., 2008; Holdefer et al., 2010; Wang et al., 2014), to our knowledge, no studies exist on the functional use of LFPs recorded from multiple microelectrodes for the selection of the optimal trajectory targeting the STN in PD. The present study demonstrates that using spectral features of LFP to identify the optimal track without any decorrelation technique provide sub-optimal results due to widely distributed neural signals and/or artifacts masking the spatially and temporally distinguishing patterns during targeting. Therefore, the LMS algorithm is used as an efficient technique to decorrelate the tracks by keeping localized activities in each. The adaptive LMS algorithm is widely used in the biosignal processing field since early 80s for signal enhancement due to its efficiency and low complexity (Widrow et al., 1975; Ferrara and Widrow, 1982; Chen et al., 1990). Since the decorrelation is being done recursively without violating the causality constraint, where each channel is predicted by the current samples of other data channels, the algorithm can be easily executed on standard PC architectures in real-time. Since it is an adaptive technique, the time and depth varying parameters allows tracking time and depth varying LFP activity and does not suffer from the cross talk as much as in the common average based derivation. It should be noted that since it estimates current signal by using a linear combination of other signals, the LMS algorithm cannot fully eliminate the high amplitude artifacts if they are not distributed among the tracks, which constitutes a major drawback of the algorithm. One way of reducing the effect of large artifacts and keep the system stable is to use an error threshold with upper and lower boundaries. Another important factor influencing the benefit of the algorithm is the learning parameter μ. It should be investigated by considering signal properties such that adaptation of the system should be neither very slow nor very fast.

Spectral analysis showed that beta oscillations are getting stronger as the electrodes approach the STN. Not only beta oscillations but also strong HFOs can be observed in the STN area well aligned with beta oscillations. This strong relation is noted in the tracks selected by neurosurgeons in 17 recordings out of 25. In rest of the eight recordings, HFO was either weak or fully absent or they were noted only in one of the un-selected tracks. The energy changes above and in the STN were used to localize the dorsal border. The RMS error of prediction for the dorsal border of STN is obtained from 1.18 to 1.98 mm when the different features are used. The minimum prediction error is found with the power of HFOs (1.18 mm) indicating that, despite the unknown functional role of these high frequency components, they can still be eligible in STN targeting. The LFP is a continuous process and does not suffer from SUA isolation challenge while the target variable is a SUA driven information which is also prone to interpretation error and isolation challenge. Considering the dorsa-ventral size of STN, 1.22 mm prediction error in depth with the fused features may represent 11% in DBS electrode 3387 or 16% in 3389 difference which can be easily compensated with the multiple contacts of the DBS electrode.

The features computed above the STN border provided poor results in prediction of optimal track. We note that the optimal track can be predicted with higher accuracy with the features obtained below the dorsal border of STN. Analysis manifest that 14 recordings out of 25 (56% of entire dataset) indicate spatially distinct HFOs together with beta activity above the dorsal border of STN (see Figures 7C,D) in at least one of the un-selected tracks. It can be assumed that these relatively weak oscillations located away from the dorsal border of STN are recorded from thalamic structures. To our knowledge, this considerable amount of thalamic oscillations in PD are not well studied phenomena. These findings presented here might be used as spatial markers in STN localization and might form the basis of further investigation into PD pathophysiology from a spatio-spectral perspective.

It should be noted that our classification technique could not predict the selected track in 20% of the subjects. We did not observe a gender difference between these five misclassified subjects. Specifically, three of them were men and two of them were women. None of the misclassified patients were tremor dominant. Three of these patients were typical PD and the other two were bradykinetic/rigid. The mean age and disease duration were 62.2 ± 13.6 and 12 ± 4.6, respectively and were not significantly different from other correctly classified patients (62.1 ± 8.3 and 10.1 ± 4.6, respectively). The misclassification in the 20% of the patients occurred due to many different factors in LFP signal including weak activity or similar activity between tracks. During these recordings, we did not use any sedation. Therefore, weak activity cannot be related to anesthesia. In one patient with typical PD phenotype, the LFP signal was weak across all tracks. We noted that the beta and HFO activity started to develop toward the bottom border of the planned target deeper than the other patients. We believe that in this particular patient the weak activity across all tracks can be described with the electrode positions. Our observations indicate that the three tracks just started to enter the STN and did not fully went through it. In another misclassified case, the LFP activity was quite strong and similar in two out of three tracks. Therefore, the classifier output was very close for these two tracks. In the other three patients, the HFO activity in the SUA selected track was weak compared to LFP selected track. Studies hypothesize that maximum beta band (13–32 Hz) and gamma band (48–220 Hz) power is highly correlated with stimulation programming parameters in DBS chronic electrode (Ince et al., 2010). When a particular contact pair on the electrode shows strong beta and gamma oscillations, it's assumed that the electrode is closer to the source so that lower stimulation would provide better symptom improvement and less side effects. The present study supports these results. We observed higher stimulation voltages in those patients where the LFPs did not correlate with MER/SUA selected tracks. Despite the lack of statistical significance, the stimulation voltages in the 6-mo-programming of implantable pulse generator (IPG) indicate lower values in the patient group having stronger LFPs in beta and/or HFO bands. A study with larger sample size would be needed to test the validity of this observation.

## Conclusion

The present study describes an automated approach for electrophysiological localization of STN, using microelectrode-recorded LFPs acquired during DBS surgery simultaneous to MER. This work is novel, in that it is the first study to combine different sub-band features derived from beta (11–32 Hz) and HFOs (150–450 Hz) of LFPs in order to (1) estimate the optimal track for DBS implantation, and (2) identify the dorsal STN border, with high accuracy. This work also contributes to knowledge about the neurophysiology of PD by describing the spatial localization of HFOs. Because recording LFPs *simultaneous* with MER/SUA does not prolong the total duration of surgery, using this technique online in the operating room would increase the chance of optimal placement of the DBS macroelectrode within the motor sub-territory of the STN, without an appreciable downside. Fused with existing mapping techniques, automated online LFP analysis may increase the accuracy of the DBS macroelectrode placement. This might contribute to the efficacy of DBS by reducing the stimulation voltage and associated side effects. Since the electrode placement is guided by LFP activity, the current technique could also be useful to monitor the LFP events which are capable to fine tune the future DBS settings in a closed loop paradigm (Ince et al., 2010; Rouse et al., 2011; Priori et al., 2013).

Further prospective investigations regarding the clinical outcomes using this technique of optimal track selection are warranted.

## Author contributions

NI and IT designed the study, collected the data, wrote the manuscript, and conducted the analyses. AA and AV performed the surgeries, contributed to data collection, and interpretation of the results. JJ performed the behavioral tests during surgery, evaluated the condition of subjects, and contributed to interpretation of the results. All authors reviewed the manuscript and approved the final version of the manuscript.

### Conflict of interest statement

The authors declare that the research was conducted in the absence of any commercial or financial relationships that could be construed as a potential conflict of interest.
